# Impact of intrinsic and extrinsic religiosity on cannabis use in adolescents: A structural equation modelling approach to data from the National Survey on Drug Use and Health (NSDUH) 2015–2019

**DOI:** 10.1192/j.eurpsy.2024.420

**Published:** 2024-08-27

**Authors:** G. Carrà, F. Bartoli, A. Canestro, C. A. Capogrosso, P. E. Bebbington, C. Crocamo

**Affiliations:** ^1^Department of Medicine and Surgery, University of Milano-Bicocca , Monza, Italy; ^2^Division of Psychiatry, University College London, London, United Kingdom

## Abstract

**Introduction:**

Religiosity is believed to be a factor that may reduce the risk of addiction and substance use both in adults and in young people. It is a complex construct that is neither measurable nor objectifiable, thus it must be estimated from proxy characteristics. For this purpose, researchers differentiate between subjective religiosity (i.e., individual religious experience) and extrinsic religiosity, that is, participation to religious services (extrinsic-personal subtype) or to social activities consistent with religion-based principles (extrinsic-social subtype).

**Objectives:**

This work aimed at exploring the role of different facets of religiosity – intrinsic (subjective), extrinsic-personal (service attendance), and extrinsic-social (church-based social activities) – in terms of deterring cannabis use among adolescents.

**Methods:**

Aggregated data of NSDUH (2015-2019) on 68,263 adolescents between 12 and 17 years of age were analysed using a structural equation modelling (SEM) to determine pathways of intrinsic and extrinsic components of religiosity in cannabis use. Several covariates were considered in the analyses, including comorbid depression and civil volunteering activities.

**Results:**

About 15% of participants admitted cannabis use in the previous year. Intrinsic and extrinsic-personal religiosity was reported by 66% and 25% of the sample, respectively. A percentage of fifty-seven of participants were involved in at least one faith-based activity, while 74% reported participation in secular community activities. Both intrinsic and extrinsic-personal religious components were likely to reduce cannabis use at the SEM regression model analysis controlling for putative confounders (cannabis use coeff.: -0.065, p=0.001; coeff.: -0.176, p<0.001, respectively). Considering the joint contribution of relevant covariates (community-based activities, lifetime MDE, sex, and poverty status), the outputs were similar. Cannabis use was not influenced by extrinsic-social component of religiosity, even though the involvement in non-faith based volunteering activities was protectively associated.

**Conclusions:**

From a policy-makers perspective, the reduction of cannabis use among young people may be obtained by supporting secular volunteering programs, which seem to be a cost-effective strategy. Moreover, whilst promoting religiosity is beyond the scope of any preventive programs, religious practices should be considered relevant protective factors.

**Disclosure of Interest:**

None Declared

